# Differentiating solitary brain metastases from glioblastoma by radiomics features derived from MRI and 18F-FDG-PET and the combined application of multiple models

**DOI:** 10.1038/s41598-022-09803-8

**Published:** 2022-04-06

**Authors:** Xu Cao, Duo Tan, Zhi Liu, Meng Liao, Yubo Kan, Rui Yao, Liqiang Zhang, Lisha Nie, Ruikun Liao, Shanxiong Chen, Mingguo Xie

**Affiliations:** 1 School of Medical and Life Sciences, Chengdu University of Traditional Chinese Medicine, Chengdu, China; 2 College of Computer & Information Science, Southwest University, Chongqing, China; 3 Department of Radiology, Chongqing Traditional Chinese Medicine Hospital, Chongqing, China; 4 Department of Radiology, The First Affiliated Hospital of Chongqing Medical University, Chongqing, China; 5 GE Healthcare, MR Research China, Beijing, China; 6 Department of Radiology, Chongqing General Hospital, Chongqing, China; 7 Department of Radiology, Hospital of Chengdu University of Traditional Chinese Medicine, Chengdu, China

**Keywords:** Cancer, Oncology

## Abstract

This study aimed to explore the ability of radiomics derived from both MRI and 18F-fluorodeoxyglucose positron emission tomography (18F-FDG-PET) images to differentiate glioblastoma (GBM) from solitary brain metastases (SBM) and to investigate the combined application of multiple models. The imaging data of 100 patients with brain tumours (50 GBMs and 50 SBMs) were retrospectively analysed. Three model sets were built on MRI, 18F-FDG-PET, and MRI combined with 18F-FDG-PET using five feature selection methods and five classification algorithms. The model set with the highest average AUC value was selected, in which some models were selected and divided into Groups A, B, and C. Individual and joint voting predictions were performed in each group for the entire data. The model set based on MRI combined with 18F-FDG-PET had the highest average AUC compared with isolated MRI or 18F-FDG-PET. Joint voting prediction showed better performance than the individual prediction when all models reached an agreement. In conclusion, radiomics derived from MRI and 18F-FDG-PET could help differentiate GBM from SBM preoperatively. The combined application of multiple models can provide greater benefits.

## Introduction

Glioblastoma (GBM) and metastatic tumours (MET) account for a large proportion of brain tumours, especially in the elderly^1^. The differentiation of GBM and MET faces great challenges in imaging diagnosis because of the sharing imaging features, such as cystic necrosis, ring enhancement, and obvious peripheral oedema, and some METs appear solitary, while GBM may sometimes be multifocal^2,3^. In particular, it is challenging to differentiate MET from GBM, while MET appears as SBM. However, the differentiation necessary for the treatment of these two tumours is entirely different^4^. Histopathological examination is still the gold standard for qualitative diagnosis. However, the accuracy of pathological diagnosis will also be affected by various factors^5,6^. Sometimes, a biopsy is unavailable for specific reasons, such as the patient being too weak to undergo surgery, the tumour being involved, or being too close to an eloquent area. Therefore, noninvasive and highly accurate differential diagnosis methods are of great significance.

MRI plays a vital role in distinguishing brain GBM from brain SBM^7,8^; however, it is not very practical by traditional research methods based on qualitative features and parameters from MRI^9^. Radiomics is an efficient research method for extracting many quantitative features from medical images^10,11^, providing more information than human eyes can recognize. It offers good performance in assessing the pathophysiology of tumours and distinguishing tumour characteristics^12,13^. In recent years, radiomics has made considerable progress in tumour and nontumor disease diagnosis^14–17^. There have been several studies on the differentiation of GBM and SBM by radiomics derived from MRI. For example, radiomic features extracted from peritumoral oedema areas in T1-weighted contrast-enhanced imaging (T1C) and T2-weighted imaging (T2) were used to differentiate GBM from SBM^18,19^. The above studies have shown good potential, with limited radiomics effectiveness based only on MRI. An 18F-FDG-PET examination can reflect the metabolic characteristics of tumours at the molecular level, and it plays an essential role in tumour detection, staging, and efficacy evaluation^20^. With the precision and personalization of clinical treatment, the application value of 18F-FDG-PET in tumours has been increasingly recognized and promoted. Therefore, it is necessary to incorporate 18F-FDG-PET into brain tumour radiomics research. Radiomics based on 18F-FDG-PET has been used to differentiate lymphoma and glioma of the central nervous system^21^. In a previous study by Zhang^22^, different combinations of conventional MRI (cMRI), including T1C and T2, diffusion-weighted imaging (DWI) and 18F-FDG-PET images were explored to establish different radiomic models to differentiate SBM and GBM and found that the integrated model based on cMRI, DWI, and 18F-FDG-PET had the highest discriminative power between the two tumours. However, in the clinic, advanced sequences such as DWI are not as readily available as cMRI. Therefore, we hypothesize that the radiomics features derived from cMRI and 18F-FDG-PET can also better differentiate the two tumours than MRI alone. Some previous studies on radiomics have shown that each classifier has advantages and limitations^23,24^. It is difficult to choose an absolute optimal model. Therefore, we hope to build a variety of models and jointly apply these models to obtain greater benefits.

## Materials and methods

### Study population

We retrospectively collected the imaging data of brain tumours in 100 patients (50 SBMs, 50 GBMs) who underwent MRI and 18F-FDG-PET/CT scanning in the First Affiliated Hospital of Chongqing Medical University from April 1, 2016, to March 10, 2021. This study complies with the Declaration of Helsinki, and research approval was granted from the Biomedical Research Ethics Committee of Chongqing Medical University with a waiver of research-informed consent. To avoid the inconsistency of the acquired image acquisition and scanning parameters, which may affect the radiological characteristics and quantitative analysis^25^, the MRI images of all cases were acquired from only one MRI scanner and the same as the PET.

The inclusion criteria of patients were as follows: (a) glioblastoma or metastasis confirmed by surgery and pathology; (b) preoperative cranial MR imaging, including T2 and T1C, and preoperative cranial 18F-FDG-PET examination; and (c) the interval between preoperative MRI examination and 18F-FDG-PET examination was no more than two weeks. The exclusion criteria were as follows: (a) multiple tumours; (b) a history of brain tumour biopsy or treatment before MRI and 18F-FDG-PET examination; and (c) unqualified image quality with artefacts or tumour size less than 1 cm. The patient selection process flowchart is shown in Fig. S1.

### MRI/18F-FDG-PET protocol

MR images were obtained from the 3.0 T MRI system (Signa HDXT, GE Healthcare, Milwaukee, USA) with an 8-channel head coil. The main parameters of the T1C sequence were as follows: repetition time (TR) = 750 ms, echo time (TE) = 15 ms, slice thickness = 5 mm, and slice interval = 1 mm. The main parameters of the T2 sequence were as follows: TR = 8,000 ms, TE = 140 ms, flip angle = 90°, slice thicknesses = 5 mm, and interval = 1 mm.

A PET/CT scanner (Philips Gemini TF 64 PET/CT scanner) was used for 18F-FDG PET data acquisition. The participants fasted for at least 4 h before 18F-FDG (produced by Sumitomo accelerator of Japan with a radiochemical purity of > 95%), administered injection intravenously at a dose of 5.55 MBq/kg and then rested in a quiet, dim room for 40–60 min before PET/CT scanning. A PET/CT scan of the head was performed for a one-bed position (5 min/bed position) with a slice thickness of 2 mm. The 18F-FDG-PET images acquired from the PET/CT system were calibrated on the PET/CT workstation, on which the interpolation of the 18F-FDG-PET image in DICOM format was performed to double the physical resolution of the image.

### Image preprocessing and segmentation

First, MRI and 18F-FDG-PET data were imported into DicomBrowser software (https://nrg.wustl.edu/software/dicom-browser) for data desensitization, and the desensitized images were loaded into 3D-Slicer (version 4.11, https://www.slicer.org) for registration. T1C and 18F-FDG-PET images were registered separately based on the T2 images. A radiologist with 5 years of experience delineated the tumour and the oedema area around the tumour on the T2 images. After all delineations were complete, a neuroimaging doctor with 10 years of experience modified and determined the final delineated area. The region of interest (ROI) was copied to the corresponding layers of the registered T1C and 18F-FDG-PET images. In this way, the mask data for each of the three sequences were formed. The two doctors were unaware of the pathological types of all cases.

### Feature selection and model building

For all MRI data, the hybrid white-stripe method was used to perform signal intensity normalization to avoid data heterogeneity bias^26^. Referring to the Image Biomarker Standardization Initiative (IBSI), the radiomics features of T2, T1C, and 18F-FDG-PET were obtained by using Python's PyRadiomics package. All features were extracted from the original and derived images. The latter was processed by a wavelet filter (Wavelet) and Laplacian of Gaussian filter (LoG). The t test was performed on the features extracted from the GBM and SBM cases to eliminate features with no significant difference. The features selected by t test were then used to determine effective features using five dimensionality reduction methods as follows: linear discriminant analysis (LDA), principal component analysis (PCA), partial least squares regression (PLS), near-collar component analysis (NCA), and least absolute shrinkage and selection operator (LASSO)^27^. Both LDA and PCA are linear dimensionality reduction methods that transform the original n-dimensional dataset into a new dataset through an orthogonal transformation. The partial least squares method uses the basic relationship between the independent and dependent variables to model the covariance structure in the two-variable space to achieve dimensionality reduction. NCA uses the Mahalanobis distance as the distance measurement. The conversion matrix was obtained through the dimensionality reduction in original data and learned by continuously optimizing the classification accuracy. LASSO dimensionality reduction uses the L1 regularization linear regression method to perform dimensionality reduction and to zero part of the learned feature weight, thereby achieving feature sparseness and reducing the data dimensionality. Five classification algorithms were chosen: support vector machine (SVM), logistic regression (LR), K nearest neighbours (KNN), random forest (RF), and adaptive boosting (AdaBoost). SVM classification performance is excellent in a small sample of machine learning tasks^28^. The logistic regression (LR) classifier runs faster and has higher requirements for feature engineering^29^. The idea of the KNN classification algorithm is simple and effective, but there is also a large number of calculations during the classification process, which requires considerable memory^30^. Random forest (RF) reduces the risk of overfitting by averaging decision trees. It is virtually a stable classification method, but the calculation is complex and requires more time to train the model^31^. Adaptive boosting (AdaBoost) is a vital ensemble learning technology that enhances a weak learner with a prediction accuracy only slightly higher than random guessing into a strong learner with higher prediction accuracy^32^. However, the disadvantage of this classifier is that it is more sensitive to outliers.

The entire dataset was split into a training cohort (GBM: n = 39, SBM: n = 41) and a validation cohort (GBM: n = 11, SBM: n = 9) by stratified sampling using computer-generated random numbers at a ratio of 8:2, and 25 models were generated by applying fivefold cross-validation with the five dimensionality reduction methods and the five classification algorithms. Nomenclature was adopted by combining the names of the dimensionality reduction method and the classification algorithms, e.g., "LASSO_LR": a combination of the LASSO dimensionality reduction method and the LR classification algorithms. Three model sets were built, in which 25 models with 18F-FDG-PET and MRI data were regarded as the integration set, 25 models with isolated MRI data were regarded as the MRI set, and 25 models with 18F-FDG-PET data alone were regarded as the PET set.

### Individual and combined application of models

After the three model sets were built, the receiver operating curve (ROC) of each mode was drawn, and the area under the receiver operating curve (AUC) was also calculated. The differences in the average AUC of the three model sets were compared. The model set with the highest average AUC was selected and then ranked the 25 models according to the AUC level. To verify the performance stability of models of different AUC levels, 15 models with three levels of AUC were selected and equally divided into three groups. To present a certain level of difference in AUC value between the models of the three groups, we defined 5 models with the AUC ranking of 1-5th as Group A, 5 models ranked 11-15th as Group B, and 5 models ranked 21-25th as Group C.

Individual and combined application of five models in the three groups were performed. The same weighting and a simple majority vote method^33^ were used to explore the combination performance of the five models in each group. During this process, each model was regarded as a specialist and provided with the same weight in the diagnosis. The final diagnosis was made according to the simple majority rule^34,35^. For instance, a case was determined to be GBM when more than three of the five models predicted it to be GBM. According to the consistency of voting results, three agreement patterns were obtained: 3A pattern referring to 3 models reaching an agreement that a case was predicted as GBM or SBM by three of the five models; 4A pattern referring to 4 models reaching an agreement; 5A pattern referring to 5 models reaching an agreement. Accuracy, sensitivity, and specificity were used to evaluate the performance of individual and joint voting prediction.

**Figure 1:**
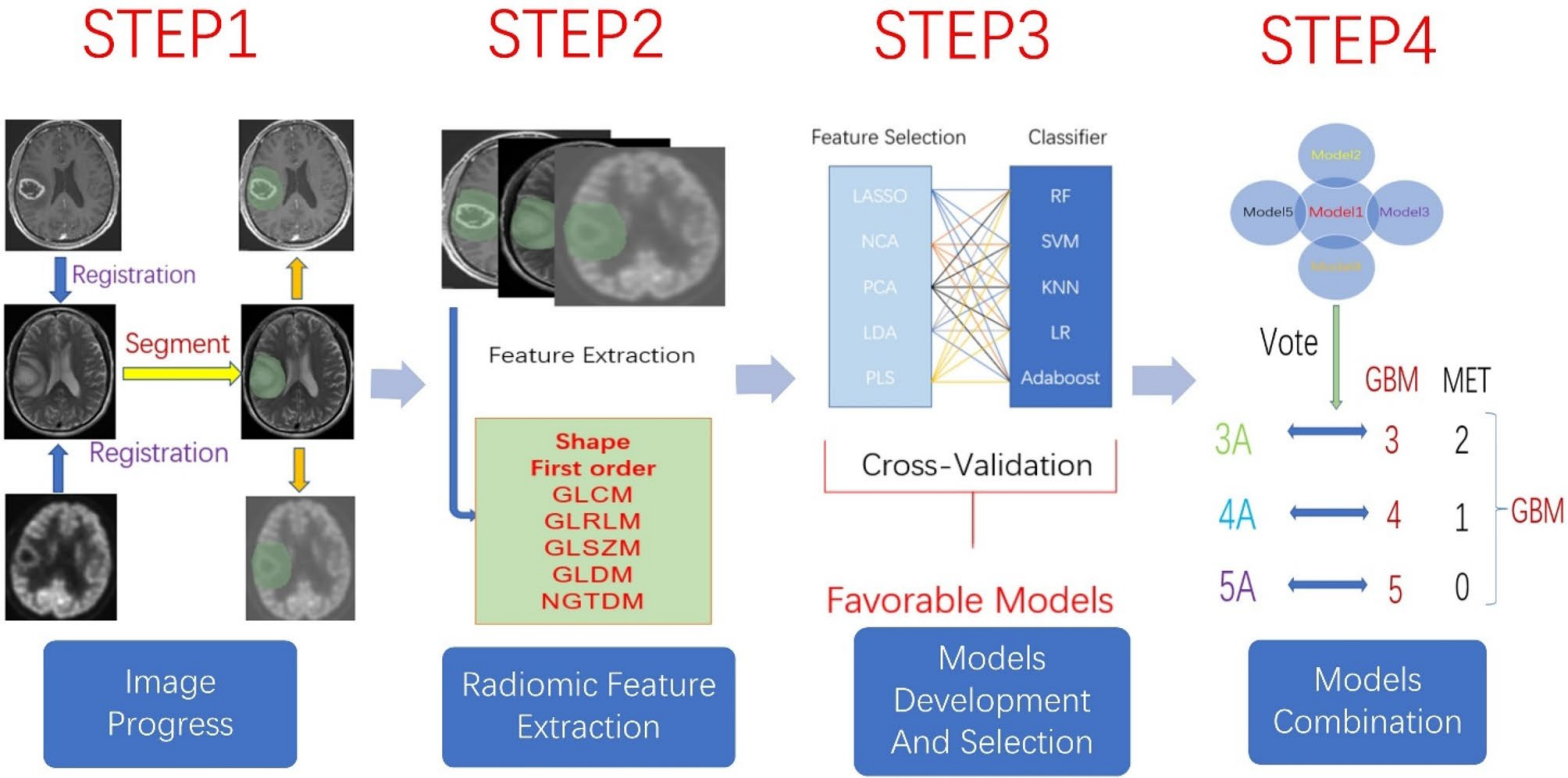
Workflow of current study. (1) The expert segment the region of interest on the image. (2) Radiomic features were extracted for further analysis. (3) Five feature selection methods and five classifiers combined into twenty-five models with the help of cross-validation in the training cohort. Part of the model was picked out and divided into three groups, five models in each group. (4) Combined application of the five models through voting strategies within the group.

The entire workflow of our research is shown in Fig. 1.

### Statistical analysis

Pearson's chi-square test was used to compare the sex difference between GBM and SBMS in the entire data, training cohort, and validation cohort. Student's t test was applied to compare the age difference between GBM and SBM. The Mann–Whitney U test was used to compare the differences in the distribution of AUC between each two of the three model sets. All statistical analyses above were carried out with SPSS 19.0 statistical software (https:www.ibm.com/products/spss-statistics). Delong’s test was performed with Python 3.8 (https://www.python.org/downloads/release/python-380) for the difference in the AUC values of the models. All statistical tests were two-sided, and the statistical significance level was set at 0.05. P values of less than 0.05 were considered to be statistically significant.

### Statement

This study complies with the Declaration of Helsinki, and research approval was granted from the Biomedical Research Ethics Committee of Chongqing Medical University with a waiver of research-informed consent.

## Results

No significant difference between GBM and SBM in age and gender was found in the entire data, the training and validation cohort in individual and joint voting prediction. No significant differences were found between GBM and MET in anatomical characteristics, necrosis appearance, or oedema appearance. See Table 1 for details.

**Table 1:** Clinical characteristics of the entire data and the sub-dataset.

	Entire data			Training cohort			Validation cohort		
	GBM (n = 50)	SBM (n = 50)	*P*	GBM (n = 39)	SBM (n = 41)	*P*	GBM (n = 11)	SBM (n = 9)	*P*
Age (years)	59.24	61.31	0.211 ^a^	58.98	61.95	0.097 ^a^	60.20	58.11	0.644 ^a^
**Sex**									
Male	26	23	0.689 ^b^	18	19	0.836 ^b^	8	4	0.409 ^b^
Female	24	27		21	22		3	5	
**Localization**									
Supratentorial	48	41	0.055 ^b^	37	34	0.182 ^b^	11	7	0.369 ^b^
Infratentorial	2	9		2	7		0	2	
**Appearance of edema**									
Yes	46	44	0.738 ^b^	36	36	0.766 ^b^	10	8	0.548 ^b^
No	4	6		3	5		1	1	
**Appearance of necrosis**									
Yes	46	43	0.522 ^b^	36	35	0.529 ^b^	10	8	0.548 ^b^
No	4	7		3	6		1	1	

*SBM* solitary brain metastases, *GBM* glioblastoma.

^a^Student ‘s t-test.

^b^Chi-square test.

Seven types of features were extracted from T2, T1C, and 18F-FDG-PET images: 2 shape-based features, 347 first-order statistical features, 413 GLCM features, 344 GLRLM features, 288 GLSZM features, 285 GLDM features, and 62 NGTDM features. Shape-based features were extracted from the original image, and other features were extracted from both the original and the derived images processed by a filter on the original image (Table 2).

**Table 2:** Extracted features of image data after the t-test.

Sequence	Firstorder	GLCM	GLRLM	GLSZM	GLDM	NGTDM	Shape	ALL
PET	63	86	49	46	44	20	0	308
T1c	96	111	124	107	101	9	1	549
T2	188	216	171	135	140	33	1	884
SUM	347	413	344	288	285	62	2	1741

*GLCM* grey-level co-occurrence matrix, *GLRLM* grey-level run length matrix, *GLSZM* grey-level size zone matrix, *GLDM* grey-level dependence matrix, *NGTDM* neighboring grey tone difference matrix.

**Figure 2:**
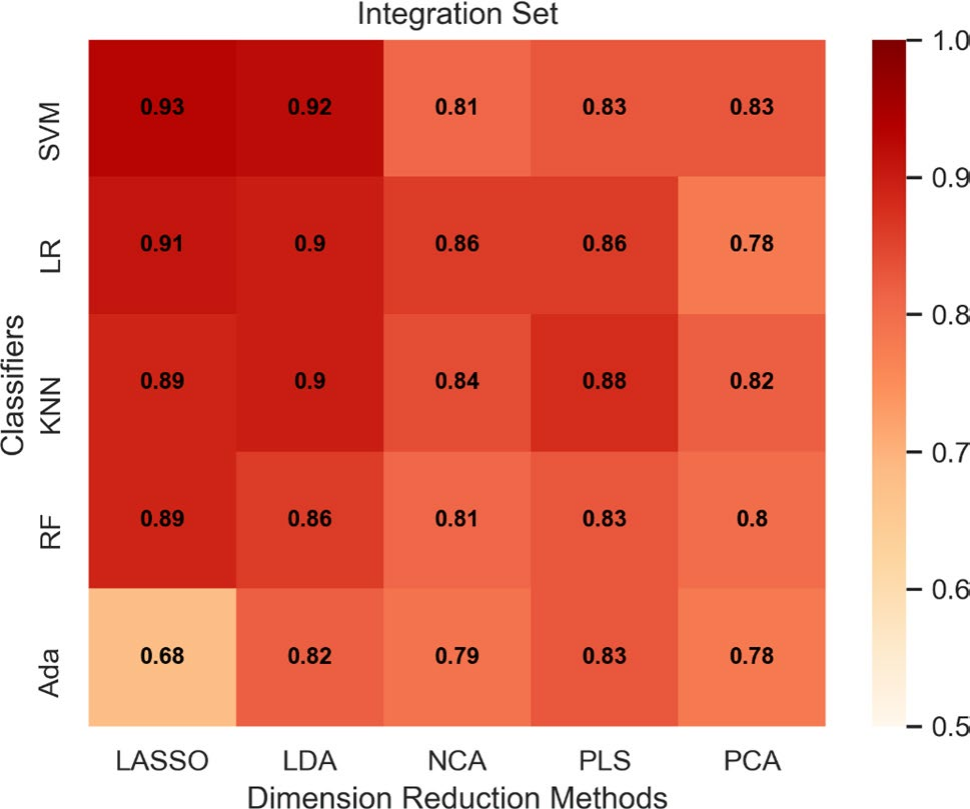
The heat map of Fivefold mean AUC of Integration Set in validation cohort. Created by python3.8 (https://www.python.org/downloads/release/python-380).

**Figure 3:**
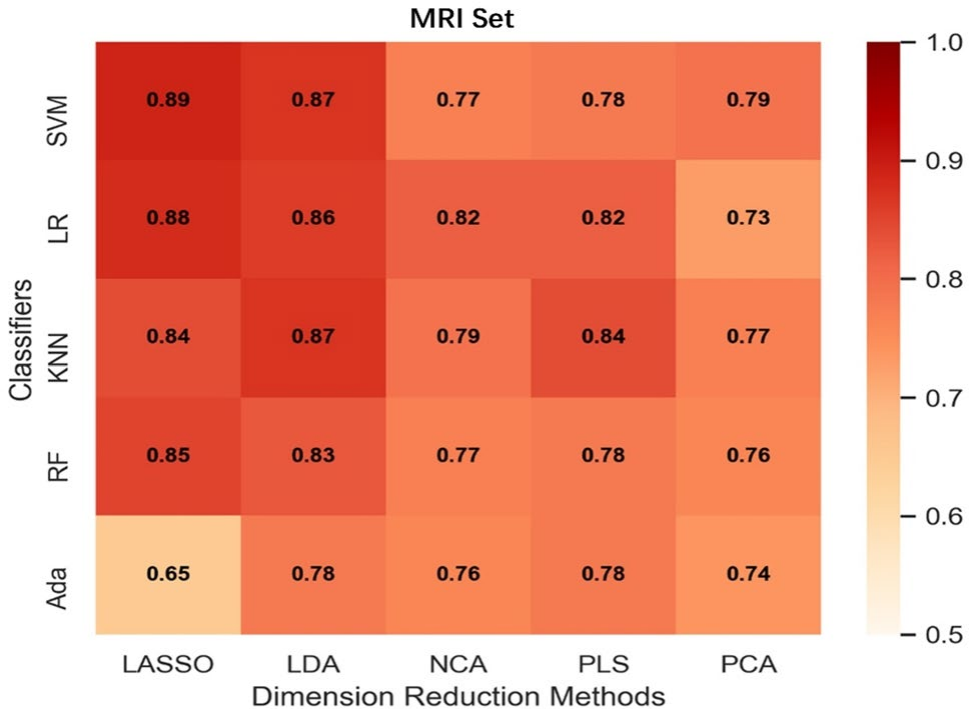
The heat map of Fivefold mean AUC of MRI Group in validation cohort. Created by python3.8 (https://www.python.org/downloads/release/python-380).

**Figure 4:**
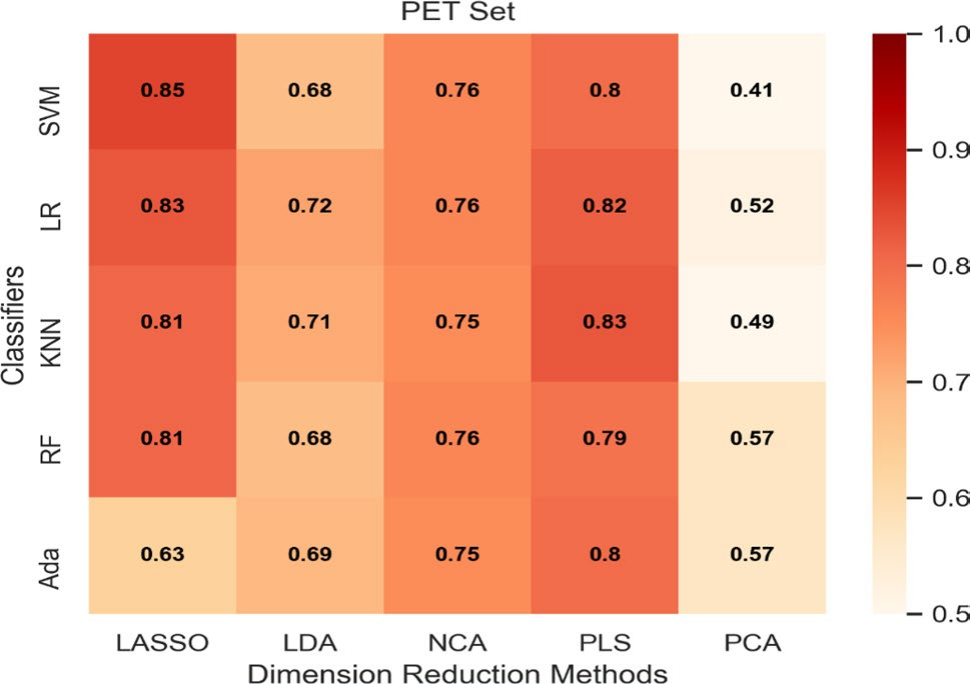
The heat map of Fivefold mean AUC of PET Group in validation cohort. Created by python3.8 (https://www.python.org/downloads/release/python-380) .

AUC heatmaps of the integration set, MRI set, and PET set are shown in Figs. 2, 3 and 4. The average AUC of the integration set was 0.84, that of the MRI set was 0.80, and that of the PET set was 0.71. Significant differences were found (P < 0.05) between the integration set and MRI set (P = 0.008), the integration set and PET set (P = 0.000005), and the MRI set and PET set (P = 0.003). In Figs. 2, 3 and 4, we found that each of the 25 models in the integration set had a higher AUC value than the MRI and PET sets. The results of pairwise comparisons of AUC values for all models in the three model sets are shown in Table S2. The pairwise comparisons of the models with the highest AUC in each of the three model sets were as follows: integration set vs. MRI set (0.93 vs. 0.89, P = 0.048), integration set vs. PET set (0.93 vs. 0. 85, P = 0.013), MRI set vs. PET set (0.89 vs. 0.85, P = 0.059).

**Figure 5:**
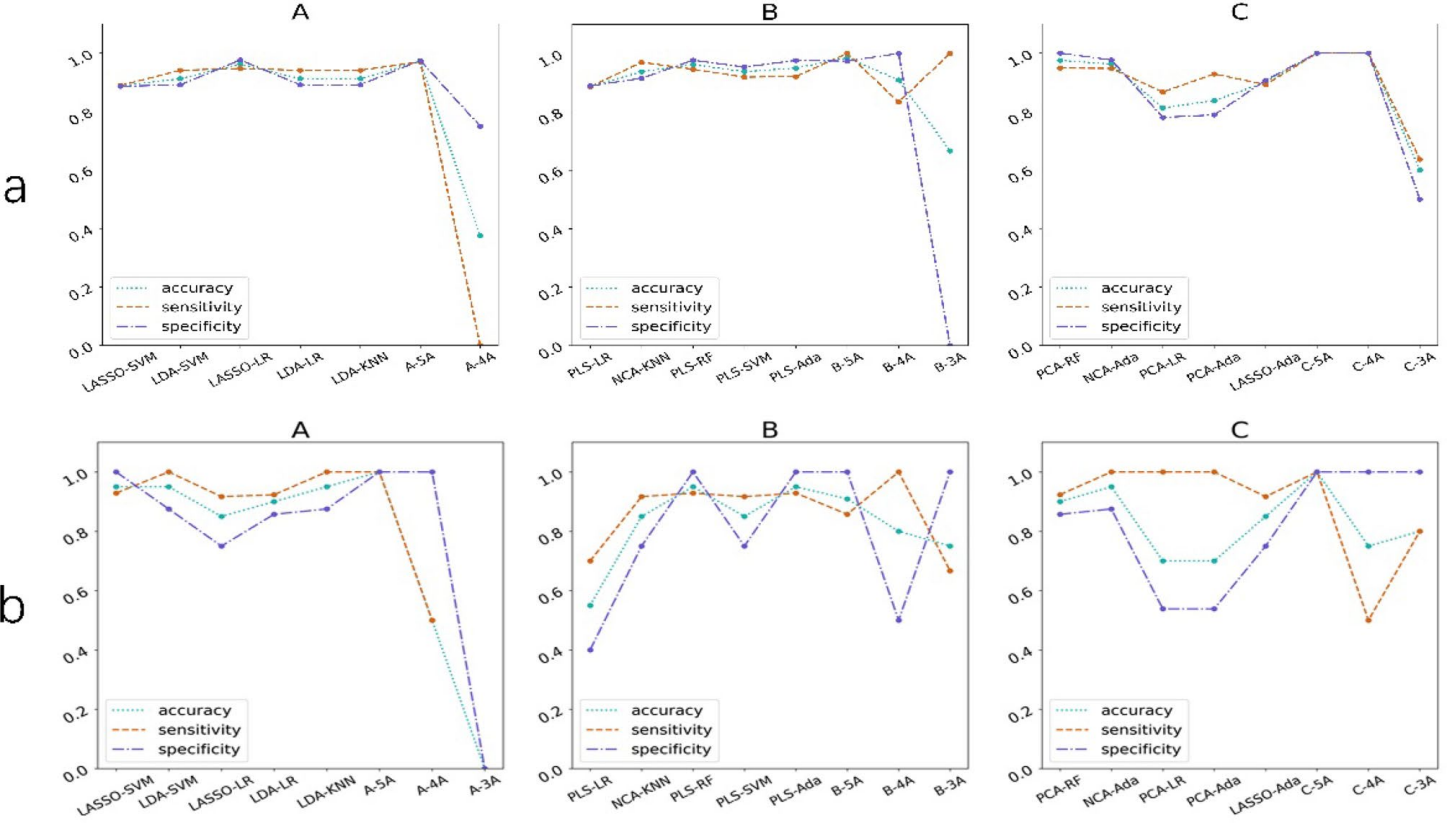
The performance of the five individual models and the combined use of each group: (**a**) the performance in training cohort; (**b**) The performance in training cohort. A, group A; B, group B; C, group C. 5A, five models reach agreement; 4A, four models reach agreement; 3A, three models reach agreement.

**Figure 6:**
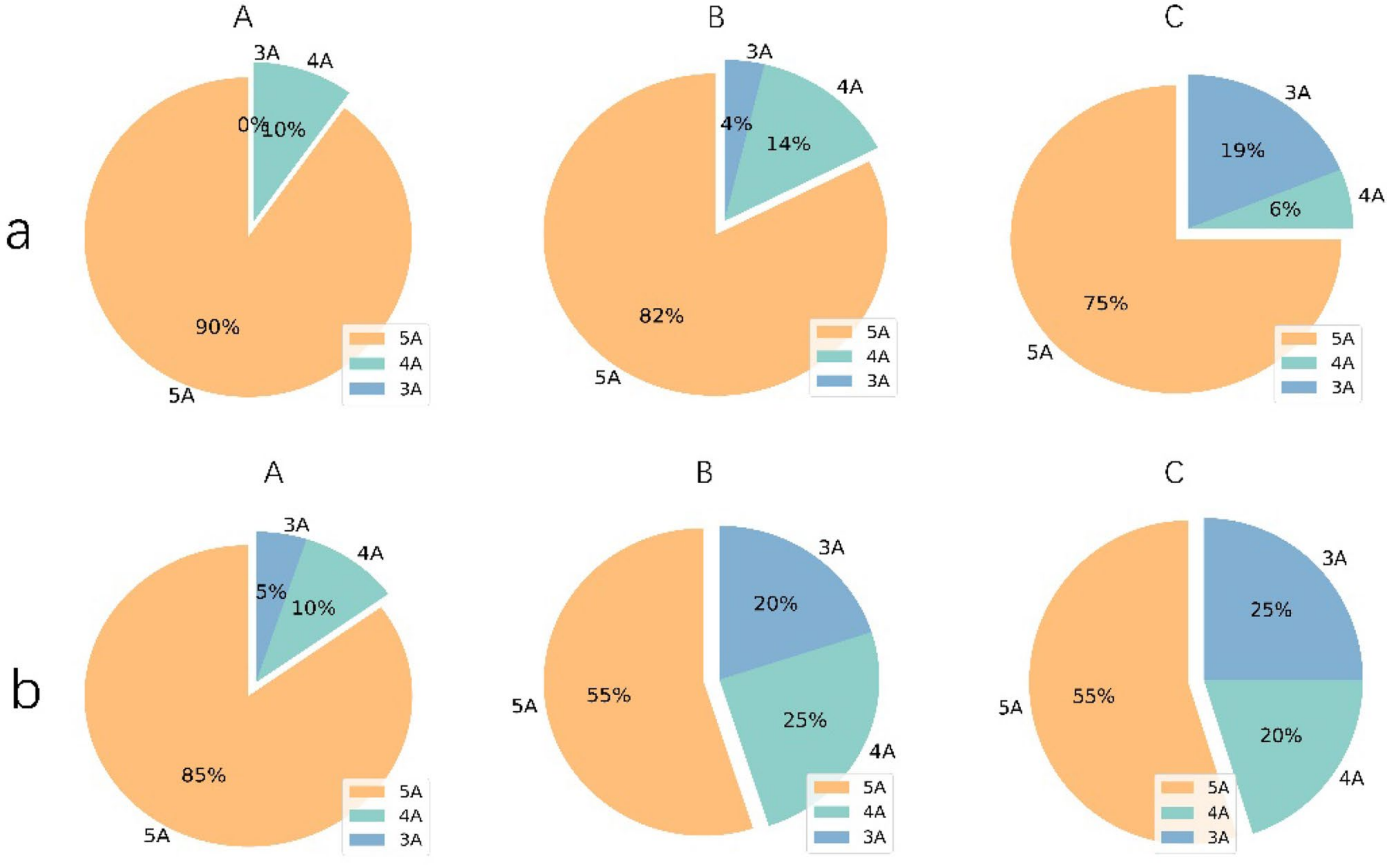
The ratios of different agreement patterns in each group: (**a**) the ratios in training cohort; (**b**) The ratios in training cohort. A, group A; B, group B; C, group C.

The integration set was finally selected with an ACC of 0.67–0.89, a sensitivity of 0.66–0.88, and a specificity of 0.65–0.92 (Table 3). Specific performance indicators of the fivefold mean value for the validation cohort of the 15 models selected from the integration set are shown in Table 3. The results of individual and joint model voting prediction in the training and validation cohorts are shown in Table S1. Compared with the individual prediction, joint model voting prediction showed that different agreement patterns had different classification performances (Fig. 5). In Group A, the 5A pattern showed the highest sensitivity, specificity, and accuracy in the training cohort(0.96, 0.97, 0.97) and the same in the validation dataset(1.0,1.0,1.0); in Group B, the 5A and 3A pattern showed the highest sensitivity(both 1.0), the 4A pattern showed the highest specificity (1.0), and the 5A pattern showed the highest accuracy (0.98) in the training cohort, the 5A, and 3A patterns showed the highest specificity (both 1.0), the 4A pattern showed the highest sensitivity (1.0), and the 5A pattern showed the highest accuracy (0.90) in the validation cohort; in Group C, the 5A and 4A patterns showed the highest sensitivity (1.0), specificity (1.0) and accuracy (1.0) in the training cohort, the 5A pattern showed the highest sensitivity (1.0), and accuracy (1.0), and the 5A, 4A and 3A patterns all showed the highest specificity (1.0) in the validation cohort. The proportions of consistent patterns in different model groups were also different (Fig. 6).

**Table 3:** The performance of the 15 selected models from Integration Set in validation cohort.

Group	Model	AUC	ACC	Sensitivity	Specificity
A	LASSO-SVM	0.93	0.83	0.76	0.92
	LDA-SVM	0.92	0.86	0.84	0.91
	LASSO-LR	0.91	0.89	0.88	0.9
	LDA-LR	0.90	0.87	0.84	0.91
	LDA-KNN	0.90	0.85	0.80	0.91
B	PLS-LR	0.86	0.78	0.76	0.82
	NCA-KNN	0.84	0.83	0.86	0.81
	PLS-RF	0.83	0.80	0.80	0.80
	PLS-SVM	0.83	0.78	0.82	0.74
	PLS-Adaboost	0.83	0.82	0.82	0.82
C	PCA-RF	0.80	0.72	0.74	0.70
	NCA-Adaboost	0.79	0.78	0.82	0.74
	PCA-LR	0.78	0.77	0.66	0.88
	PCA-Adaboost	0.78	0.77	0.70	0.84
	LASSO-Adaboost	0.68	0.67	0.68	0.65

*AUC* area under curve, *ACC* accuracy, *LASSO* least absolute shrinkage and selection operator, *LDA* linear discriminant analysis, *PLS* partial least squares regression, *NCA* near-collar component analysis, *PCA* principal component analysis, *SVM* support vector machine, *LR* the logistic regression, *KNN* K nearest neighbors, *RF* random forest, *Adaboost* Adaptive Boosting.

## Discussion

This study extended most previous radiomics studies that extracted features from cMRI sequences on enhancing tumour regions and peri-enhancing oedema regions to differentiate GBM from SBM and further incorporated 18F-FDG-PET features reflecting tumour molecular metabolism. MRI-based radiomics has been used to differentiate GBM from SBM in previous studies. Su et al.^36^, T1C-based radiomics analysis yielded AUC values of 0.82 and 0.81 in the training and validation cohorts, respectively. Ortiz et al.^37^, the AUC of the T1WI-based radiomics model was 0.896 ± 0.067. In the study of Bae et al.^19^, they extracted radiomics features from T1C tumour enhancement and T2 peritumoral oedema areas, establishing the best conventional model with an AUC of 0.89. We combined 18F-FDG-PET and cMRI features to build 25 multimodal radiomics models. Finally, the result was satisfactory in that the best model achieved an AUC value of 0.93, higher than previous studies based on cMRI alone mentioned above, although lower than the integrated model with an AUC value of 0.98 in Zhang's study^22^. In our study, the multimodal models integrating 18F-FDG-PET and cMRI improved AUC values compared to radiomics models derived from only 18F-FDG-PET and cMRI. This is consistent with our previous hypothesis that multimodal radiomics would better distinguish GBM from SBM. Unlike most previous studies in which the best model was selected from multiple models, in this study, we obtained the best model and found that the combination of multiple models was more beneficial.

In the three model sets, almost all the models using the LASSO feature selection method had higher AUC values, suggesting that LASSO is a reliable feature selection method. The classifier SVM can filter the most effective samples for the prediction task in massive feature data. In Group A of the integration set, the top 2 classifier is SVM, which proved that it also has strong generalization ability with a small data sample size. LASSO_SVM is considered the optimal model in all three model sets in our study (with an AUC of 0.93 in the integration set, 0.89 in the MRI set, and 0.85 in the PET set). This is consistent with the research results of Qian^38^, in which 84 models were built and LASSO_SVM was selected as the best model for an AUC of 0.9. The performance may be different among different models even based on the same data. Therefore, we explored the combined application value of different models instead of only choosing the best model. The combined application of multiple models is similar to multidisciplinary teamwork (MDT) in clinical practice. The collaboration between specialists in clinical practice is significant for making comprehensive and correct decisions^39,40^. In this study, the 5A pattern of the joint voting has improved accuracy, sensitivity, and specificity to varying degrees compared with the individual prediction. The performance of the 4A and 3A patterns all shows a downwards trend, and the average level of individual prediction outperformed the 3A pattern, which is similar to the results of Dong^41^. In the combined application of multiple models, it was interesting that the five models in Group A, with higher AUC values than Groups B and C, were more likely to reach an agreement (highest 5A pattern, lowest 3A pattern). In Groups B and C with lower AUC values of the models, the application of the 5A pattern improved the prediction performance more significantly. Similar results can also be found in the MRI and PET sets (Tables S3–S4 and Figs. S2–S5). Both this and previous studies^41^ have shown that the method of combining multiple models can be more beneficial, especially when the model performance is not good. The benefits of applying this method will be more obvious. Although the performance of our model is not as good as the optimal model established by Zhang’s study^22^, our study further confirms that the addition of PET features reflecting tumour metabolism can better distinguish SBM and GBM than the radiomics model based solely on cMRI features. On this basis, our study also provides a good solution for poor model performance in radiomics studies.

Of course, there are some limitations to this study. First, the image data were obtained by the same MR and PET/CT scanner. Therefore, the samples we obtained were relatively few. Although the results performed well, the generalization ability of each model still needs a large sample size for further verification. In practical work, it is difficult to obtain image data with consistent scanning parameters in different medical institutions or even in the same institution. In addition, the simple voting method with the same weight is adopted in the joint application of multiple models, and more joint application methods and comparisons with different methods can be further explored.

## Conclusion

Radiomics derived from cMRI and 18F-FDG-PET can help differentiate GBM from SBM preoperatively, which may achieve greater benefits in clinical practice. Multimodal radiomics based on MRI and 18F-FDG-PET is expected to become a powerful research method for the differentiation of intracranial tumours. The combined application of multiple models inspired by MDT can generate extra benefit, especially when the performance of the model is mediocre. The combined application of multiple models can be used as a new method in radiomics research.

## Supplementary Information

Supplementary Information.
