# Supplementary material for: Differentiating solitary brain metastases from glioblastoma by radiomics features derived from MRI and 18F-FDG-PET and the combined application of multiple models
Source: Sci Rep. 2022 Apr 6;12:5722. doi: 10.1038/s41598-022-09803-8 (PMC8986767; doi:10.1038/s41598-022-09803-8)
Supplement: Supplementary file 1 — Supplementary Information. [file 41598_2022_9803_MOESM1_ESM.docx]

Table S1: The performance of individual and joint model voting prediction on training and validation cohort in Integration Set.

| Group | Model/Patter*n* |  | Training cohort |  |  | Validation cohort |  |
| --- | --- | --- | --- | --- | --- | --- | --- |
|  |  | ACC | Sensitivity | Specificity | ACC | Sensitivity | Specificity |
| A | LASSO-SVM | 0.88 | 0.88 | 0.88 | 0.95 | 0.92 | 1.0 |
|  | LDA-SVM | 0.91 | 0.94 | 0.89 | 0.95 | 1.0 | 0.87 |
|  | LASSO-LR | 0.96 | 0.94 | 0.97 | 0.85 | 0.91 | 0.75 |
|  | LDA-LR | 0.91 | 0.94 | 0.89 | 0.90 | 0.92 | 0.85 |
|  | LDA-KNN | 0.91 | 0.94 | 0.89 | 0.95 | 1.0 | 0.87 |
|  | 5A | 0.97 | 0.96 | 0.97 | 1.0 | 1.0 | 1.0 |
|  | 4A | 0.37 | 0 | 0.75 | 0.5 | 0.5 | 1.0 |
|  | 3A | Nun | Nun | Nun | 0 | Nun | 0 |
| B | PLS-LR | 0.88 | 0.88 | 0.88 | 0.55 | 0.7 | 0.4 |
|  | NCA-KNN | 0.93 | 0.96 | 0.91 | 0.85 | 0.91 | 0.75 |
|  | PLS-RF | 0.96 | 0.94 | 0.97 | 0.95 | 0.92 | 1.0 |
|  | PLS-SVM | 0.93 | 0.91 | 0.95 | 0.85 | 0.91 | 0.75 |
|  | PLS-Adaboost | 0.95 | 0.92 | 0.97 | 0.95 | 0.92 | 1.0 |
|  | 5A | 0.98 | 1.0 | 0.97 | 0.90 | 0.85 | 1.0 |
|  | 4A | 0.90 | 0.83 | 1.0 | 0.8 | 1.0 | 0.5 |
|  | 3A | 0.66 | 1.0 | 0 | 0.75 | 0.66 | 1.0 |
| C | PCA-RF | 0.97 | 0.94 | 1.0 | 0.9 | 0.92 | 0.85 |
|  | NCA-Adaboost | 0.96 | 0.94 | 0.97 | 0.95 | 1.0 | 0.87 |
|  | PCA-LR | 0.81 | 0.86 | 0.78 | 0.7 | 1.0 | 0.53 |
|  | PCA-Adaboost | 0.83 | 0.92 | 0.78 | 0.7 | 1.0 | 0.53 |
|  | LASSO-Adaboost | 0.9 | 0.89 | 0.90 | 0.85 | 0.91 | 0.75 |
|  | 5A | 1.0 | 1.0 | 1.0 | 1.0 | 1.0 | 1.0 |
|  | 4A | 1.0 | 1.0 | 1.0 | 0.75 | 0.5 | 1.0 |
|  | 3A | 0.6 | 0.63 | 0.5 | 0.8 | 0.8 | 1.0 |

Table S2: The results of pairwise comparisons of AUC values for all models in the three model sets

| Model | Integration Set vs MRI Set  （P Value） | Integration Set vs PET Set  （P Value） | MRI Set vs PET Set  （P Value） |
| --- | --- | --- | --- |
| LASSO-SVM | 0.93vs0.89（P=0.048） | 0.93vs0.85（P=0.013） | 0.89vs0.85（P=0.069） |
| LASSO-LR | 0.91vs0.88（P=0.037） | 0.91vs0.83（P=0.025） | 0.88vs0.83（P=0.041） |
| LASSO-KNN | 0.89vs0.84（P=0.049） | 0.89vs0.81（P=0.037） | 0.84vs0.81（P=0.052） |
| LASSO-RF | 0.89vs0.85（P=0.046） | 0.89vs0.81（P=0.029） | 0.85vs0.81（P=0.058） |
| LASSO- Adaboost | 0.68vs0.65（P=0.176） | 0.68vs0.63（P=0.159） | 0.65vs0.63（P=0.394） |
| LDA-SVM | 0.92vs0.87（P=0.038） | 0.92vs0.68（P=0.001） | 0.87vs0.68（P=0.021） |
| LDA-LR | 0.90vs0.86（P=0.043） | 0.90vs0.72（P=0.003） | 0.86vs0.72（P=0.017） |
| LDA-KNN | 0.90vs0.87（P=0.057） | 0.90vs0.71（P=0.019） | 0.87vs0.71（P=0.049） |
| LDA-RF | 0.86vs0.83（P=0.044） | 0.86vs0.68（P=0.035） | 0.83vs0.68（P=0.052） |
| LDA- Adaboost | 0.82vs0.78（P=0.043） | 0.82vs0.69（P=0.029） | 0.78vs0.69（P=0.051） |
| NCA-SVM | 0.81vs0.77（P=0.146） | 0.81vs0.76（P=0.078） | 0.77vs0.76（P=0.368） |
| NCA-LR | 0.86vs0.82（P=0.532） | 0.86vs0.76（P=0.021） | 0.82vs0.76（P=0.498） |
| NCA-KNN | 0.84vs0.79（P=0.062） | 0.84vs0.75（P=0.047） | 0.79vs0.75（P=0.511） |
| NCA-RF | 0.81vs0.77（P=0.047） | 0.86vs0.76（P=0.068） | 0.77vs0.76（P=0.059） |
| NCA- Adaboost | 0.79vs0.76（P=0.276） | 0.79vs0.75（P=0.129） | 0.76vs0.75（P=0.398） |
| PLS-SVM | 0.83vs0.78（P=0.139） | 0.83vs0.80（P=0.152） | 0.78vs0.80（P=0.258） |
| PLS-LR | 0.86vs0.82（P=0.041） | 0.86vs0.82（P=0.038） | 0.82vs0.82（P=0.512） |
| PLS-KNN | 0.88vs0.84（P=0.047） | 0.88vs0.83（P=0.053） | 0.84vs0.83（P=0.296） |
| PLS-RF | 0.83vs0.78（P=0.058） | 0.83vs0.79（P=0.041） | 0.78vs0.79（P=0.213） |
| PLS- Adaboost | 0.83vs0.78（P=0.029） | 0.83vs0.80（P=0.064） | 0.78vs0.80（P=0.097） |
| PCA-SVM | 0.83vs0.79（P=0.031） | 0.83vs0.41（P=0.000） | 0.79vs0.41（P=0.004） |
| PCA-LR | 0.78vs0.73（P=0.039） | 0.78vs0.52（P=0.001） | 0.73vs0.52（P=0.008） |
| PCA-KNN | 0.82vs0.77（P=0.034） | 0.82vs0.49（P=0.000） | 0.77vs0.49（P=0.005） |
| PCA-RF | 0.80vs0.76（P=0.041） | 0.83vs0.57（P=0.001） | 0.76vs0.57（P=0.012） |
| PCA- Adaboost | 0.78vs0.74（P=0.055） | 0.78vs0.57（P=0.004） | 0.74vs0.57（P=0.017） |

Table S3: The performance of individual and joint model voting prediction on training and validation cohort in MRI Set

| Group | Model/Patter*n* |  | Training cohort |  |  | Validation cohort |  |
| --- | --- | --- | --- | --- | --- | --- | --- |
|  |  | ACC | Sensitivity | Specificity | ACC | Sensitivity | Specificity |
| A | LASSO-SVM | 0.86 | 0.75 | 0.87 | 0.8 | 0.8 | 0.77 |
|  | LASSO-LR | 0.85 | 0.69 | 0.85 | 0.78 | 0.7 | 0.84 |
|  | LDA-SVM | 0.84 | 0.81 | 0.88 | 0.8 | 0.77 | 0.9 |
|  | LDA-KNN | 0.8 | 0.81 | 0.88 | 0.8 | 0.83 | 0.73 |
|  | LDA-LR | 0.89 | 0.83 | 0.9 | 0.76 | 0.79 | 0.9 |
|  | 5A | 0.93 | 1.0 | 1.0 | 1.0 | 1.0 | 1.0 |
|  | 4A | 0.7 | 0.6 | 0.83 | 0.9 | 0.96 | 0.95 |
|  | 3A | 0.66 | Nun | 0.66 | 0.88 | 0.92 | 0.9 |
| B | PLS-LR | 0.8 | 0.9 | 0.78 | 0.6 | 0.69 | 0.5 |
|  | NCA-KNN | 0.9 | 0.94 | 0.89 | 0.8 | 0.8 | 0.8 |
|  | PCA-SVM | 0.89 | 0.92 | 0.91 | 0.85 | 0.89 | 0.9 |
|  | LDA- Adaboost | 0.83 | 0.85 | 0.8 | 0.75 | 0.85 | 0.69 |
|  | PLS-SVM | 0.9 | 0.79 | 0.92 | 0.8 | 0.78 | 0.8 |
|  | 5A | 0.98 | 1.0 | 0.97 | 1.0 | 1.0 | 1.0 |
|  | 4A | 0.98 | 1.0 | 0.97 | 0.75 | 1.0 | 0.5 |
|  | 3A | 0.78 | 0.84 | 0.88 | 0.25 | 0.33 | 0.0 |
| C | PCA-RF | 0.76 | 0.78 | 0.73 | 0.75 | 0.79 | 0.72 |
|  | PCA -Adaboost | 0.76 | 0.8 | 0.75 | 0.8 | 0.8 | 0.8 |
|  | PCA-LR | 0.78 | 0.8 | 0.8 | 0.7 | 0.8 | 0.73 |
|  | PCA-KNN | 0.77 | 0.8 | 0.77 | 0.7 | 0.78 | 0.8 |
|  | PCA-SVM | 0.8 | 0.71 | 0.88 | 0.65 | 0.71 | 0.61 |
|  | 5A | 0.95 | 1.0 | 0.98 | 0.9 | 0.95 | 0.96 |
|  | 4A | 0.9 | 0.9 | 0.8 | 0.8 | 0.79 | 0.9 |
|  | 3A | 0.7 | 0.5 | 0.7 | 0.5 | 0.5 | 0.8 |

Table S4: The performance of individual and joint model voting prediction on training and validation cohort in PET Set

| Group | Model/Patter*n* |  | Training cohort |  |  | Validation cohort |  |
| --- | --- | --- | --- | --- | --- | --- | --- |
|  |  | ACC | Sensitivity | Specificity | ACC | Sensitivity | Specificity |
| A | LASSO-SVM | 0.85 | 0.84 | 0.82 | 0.8 | 0.87 | 0.75 |
|  | LASSO-LR | 0.82 | 0.86 | 0.80 | 0.85 | 0.9 | 0.77 |
|  | PLS-KNN | 0.87, | 0.77 | 0.87 | 0.8 | 0.9 | 0.71 |
|  | PLS-LR | 0.81 | 0.84 | 0.88 | 0.7 | 0.83 | 0.64 |
|  | LASSO-KNN | 0.78 | 0.9, | 0.80 | 0.75 | 0.85 | 0.69 |
|  | 5A | 1.0 | 1.0 | 1.0 | 0.91 | 0.98 | 0.92 |
|  | 4A | 0.9 | 0.86 | 1.0 | 0.8 | 0.93 | 0.9 |
|  | 3A | 0.8 | 0.81 | 0.9 | 0.78 | 0.7 | 0.8 |
| B | NCA-LR | 0.79 | 0.88 | 0.87 | 0.78 | 0.87 | 0.75 |
|  | NCA-RF | 0.83 | 0.84 | 0.70 | 0.85 | 0.82 | 0.77 |
|  | NCA-KNN | 0.78 | 0.77 | 0.97 | 0.68 | 0.88 | 0.71 |
|  | NCA- Adaboost | 0.81 | 0.94 | 0.73 | 0.77 | 0.83 | 0.64 |
|  | LDA-LR | 0.68 | 0.7 | 0.8 | 0.72 | 0.75 | 0.69 |
|  | 5A | 1.0 | 1.0 | 1.0 | 0.91 | 0.92 | 1.0 |
|  | 4A | 0.9 | 0.95 | 0.97 | 0.8 | 0.88 | 0.9 |
|  | 3A | 0.78 | 0.89 | 0.9 | 0.75 | 0.86 | 0.7 |
| C | PCA-RF | 0.72 | 0.8 | 0.89 | 0.7 | 0.83 | 0.64 |
|  | NCA-Adaboost | 0.75, | 0.97 | 0.68 | 0.65 | 0.71 | 0.61 |
|  | PCA-LR | 0.78 | 0.75 | 0.9 | 0.7 | 0.75 | 0.66 |
|  | PCA-KNN | 0.71 | 0.87 | 0.76 | 0.65 | 0.92 | 0.61 |
|  | PCA-SVM | 0.69 | 0.79 | 0.8 | 0.65 | 0.71 | 0.61 |
|  | 5A | 1.0 | 1.0 | 1.0 | 0.85 | 0.87 | 0.98 |
|  | 4A | 0.9 | 0.8 | 0.95 | 0.80 | 0.7 | 0.83 |
|  | 3A | 0.8 | 0.78 | 0.86 | 0.78 | 0.7 | 0.66 |


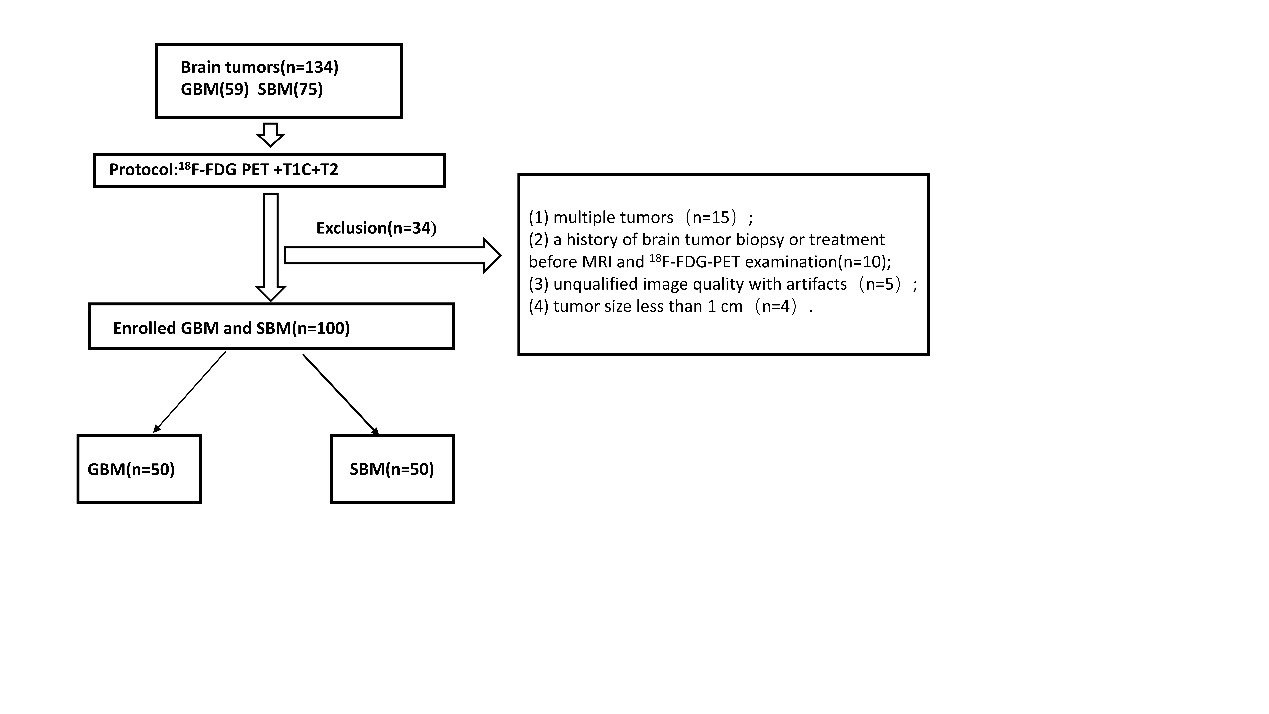


Figure S1: The flowchart of patient selection process


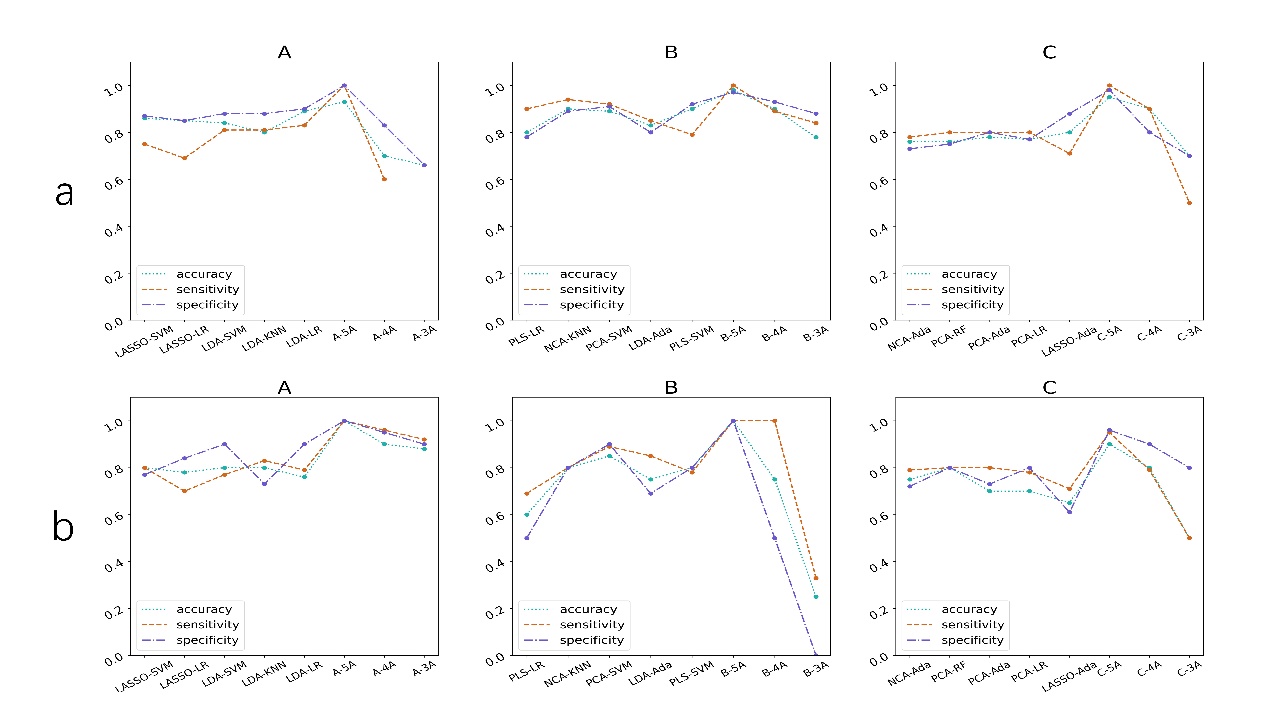


Figure S2: The performance of the five individual models and the combined use of each group in MRI Set. a, training cohort; b, training cohort. A, group A; B, group B; C, group C. 5A, five models reach agreement; 4A, four models reach agreement; 3A, three models reach agreement.


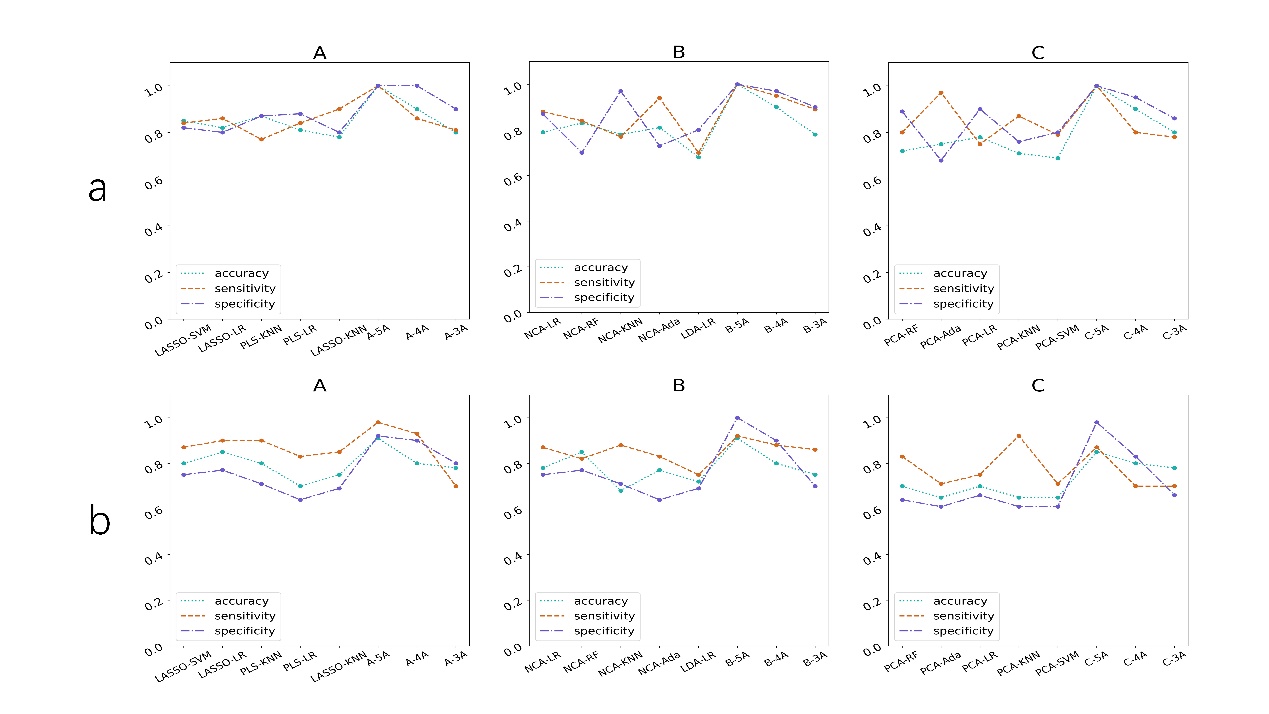


Figure S3: The performance of the five individual models and the combined use of each group in PET Set. a, training cohort; b, training cohort. A, group A; B, group B; C, group C. 5A, five models reach agreement; 4A, four models reach agreement; 3A, three models reach agreement.


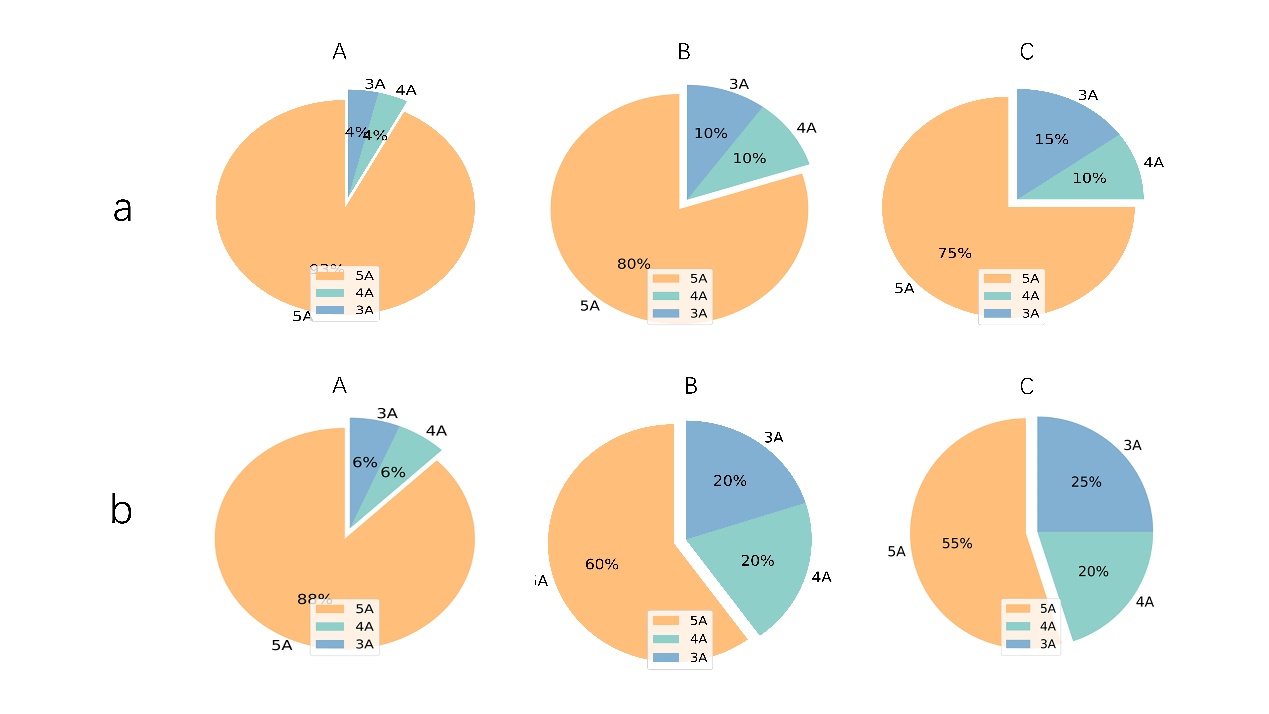


Figure S4: The ratios of different agreement patterns in each group in MRI Set. a, training cohort; b, training cohort. A, group A; B, group B; C, group C.


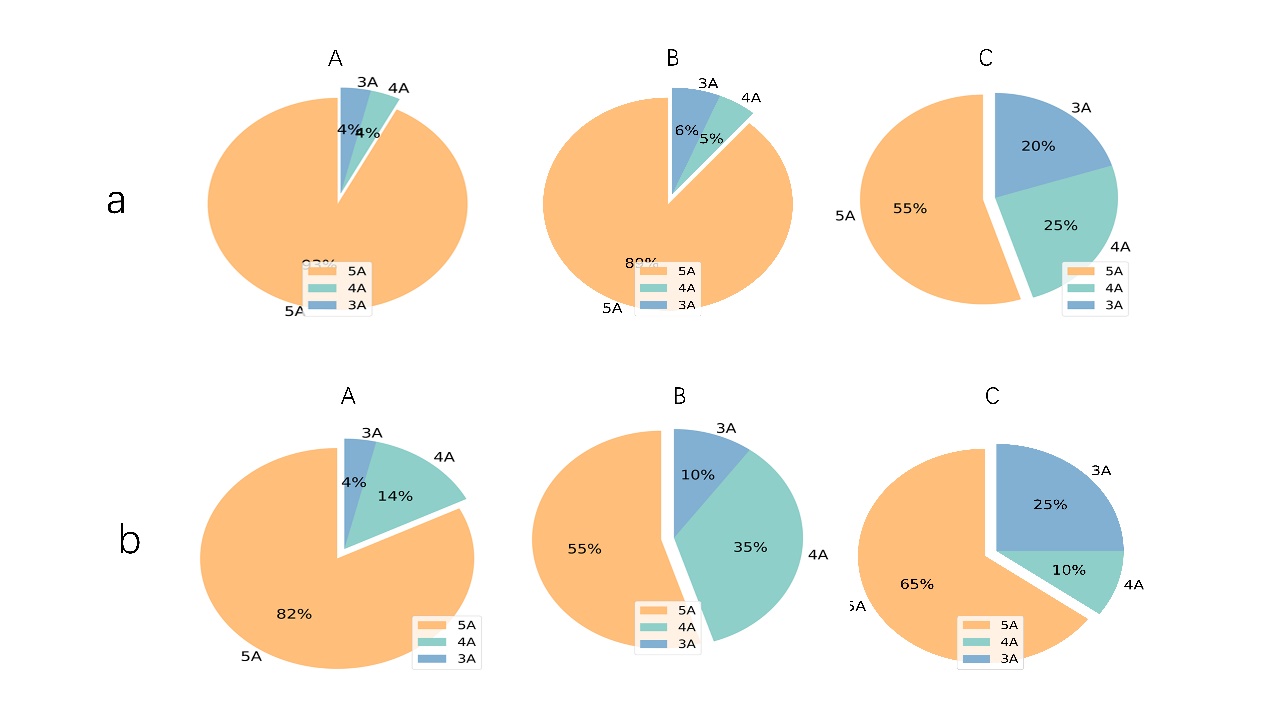


Figure S5: The ratios of different agreement patterns in each group in PET Set. a, training cohort; b, training cohort. A, group A; B, group B; C, group C.
